
JOURNAL INFORMATION
==============================
NLM Title Abbreviation: Acta Crystallogr E Crystallogr Commun
Journal ID: Acta Crystallogr E Crystallogr Commun
NLM Journal ID: 101648987
Journal ID: Acta Cryst. E

Acta Crystallographica Section E: Crystallographic Communications

EISSN: 2056-9890
Publisher: International Union of Crystallography

ARTICLE INFORMATION
==============================
PMCID: PMC10483565
PMID: 37693674
DOI: 10.1107/S2056989023006552
Article ID: wm5687
Article ID: S2056989023006552
Article version: 1
Subjects: Research Communications

Crystal structure and Hirshfeld surface analysis of 7-[(6-hy­droxy-5,5,8a-trimethyl-2-methylene­deca­hydro­naphthalen-1-yl)meth­oxy]-2H-chromen-2-one

C24H30O4

Karimli Elvin G. a b
https://orcid.org/0000-0001-8806-2975 Khrustalev Victor N. c d
https://orcid.org/0000-0003-2421-0929 Akkurt Mehmet e
https://orcid.org/0000-0002-5219-2986 Khalilov Ali N. f g
https://orcid.org/0000-0002-2648-4686 Bhattarai Ajaya h *
Aleskerova Adila N. b
Mamedov İbrahim G. g
a Azerbaijan Medical University , Bakikhanov st. 23, AZ1022, Baku, Azerbaijan
b Institute of Botany , Ministry of Science and Education of the Republic of Azerbaijan, Badamdar Highway 40, Baku, AZ1004, Azerbaijan
c Peoples’ Friendship University of Russia (RUDN University) , Miklukho-Maklay St. 6, Moscow, 117198, Russian Federation
d N. D. Zelinsky Institute of Organic Chemistry RAS, Leninsky Prosp. 47, Moscow, 119991, Russian Federation
e Department of Physics, Faculty of Sciences, Erciyes University , 38039 Kayseri, Türkiye
f ‘Composite Materials’ Scientific Research Center, Azerbaijan State Economic University (UNEC), H. Aliyev str. 135, AZ1063, Baku, Azerbaijan
g Department of Chemistry, Baku State University , Z. Khalilov str. 23, AZ1148, Baku, Azerbaijan
h Department of Chemistry, M.M.A.M.C. (Tribhuvan University) Biratnagar, Nepal
Weil M. Editor
Vienna University of Technology, Austria
Correspondence e-mail: ajaya.bhattarai@mmamc.tu.edu.np
Publication date: 2023 Sep 1
Electronic publication date: 2023 Aug 1
Volume: 79
Issue: Pt 9
First page: 777
Last page: 781
Received 2023 Jul 10; Accepted 2023 Jul 27
Copyright: © Karimli et al. 2023
Copyright year: 2023
License: This is an open-access article distributed under the terms of the Creative Commons Attribution (CC-BY) Licence, which permits unrestricted use, distribution, and reproduction in any medium, provided the original authors and source are cited.
License URL: https://creativecommons.org/licenses/by/4.0/

A full version of this article is available from Crystallography Journals Online.
Page count: 5

==============================

Acta Cryst
E
CRYSTALLOGRAPHIC COMMUNICATIONS

ISSN 2056-9890

### Crystal structure and Hirshfeld surface analysis of 7-[(6-hydroxy-5,5,8a-trimethyl-2-methylenedecahydronaphthalen-1-yl)methoxy]-2*H*-chromen-2-one

**Elvin G. Karimli,[a,b] Victor N. Khrustalev,[c,d] Mehmet Akkurt,[e] Ali N. Khalilov,[f,g] Ajaya Bhattarai,[h]* Adila N. Aleskerova[b] and İbrahim G. Mamedov[g]**

[a]Azerbaijan Medical University, Bakikhanov st. 23, AZ1022, Baku, Azerbaijan, [b]Institute of Botany, Ministry of Science and Education of the Republic of Azerbaijan, Badamdar Highway 40, Baku, AZ1004, Azerbaijan, [c]Peoples' Friendship University of Russia (RUDN University), Miklukho-Maklay St. 6, Moscow, 117198, Russian Federation, [d]N. D. Zelinsky Institute of Organic Chemistry RAS, Leninsky Prosp. 47, Moscow, 119991, Russian Federation, [e]Department of Physics, Faculty of Sciences, Erciyes University, 38039 Kayseri, Türkiye, [f]'Composite Materials' Scientific Research Center, Azerbaijan State Economic University (UNEC), H. Aliyev str. 135, AZ1063, Baku, Azerbaijan, [g]Department of Chemistry, Baku State University, Z. Khalilov str. 23, AZ1148, Baku, Azerbaijan, and [h]Department of Chemistry, M.M.A.M.C. (Tribhuvan University) Biratnagar, Nepal. *Correspondence e-mail: ajaya.bhattarai@mmamc.tu.edu.np

Received 10 July 2023
Accepted 27 July 2023

Edited by M. Weil, Vienna University of Technology, Austria

**Keywords:** crystal structure; *Ferula persica*; hydrogen bonds; C—H⋯π interactions; Hirshfeld surface analysis.

**CCDC reference**: 2211???

**Supporting information**: this article has supporting information at journals.iucr.org/e

The title compound, $C_{24}H_{30}O_4$, consists of two *trans*-fused cyclohexane rings attached to a chromen-2-one moiety through an oxymethylene bridge. The cyclohexane rings adopt half-chair and chair conformations. In the crystal, molecules are linked by C—H⋯O and O—H⋯O hydrogen bonds, forming a triperiodic network. Furthermore, C—H⋯π interactions stabilize the crystal structure. A Hirshfeld surface analysis revealed that the most important contributions to the crystal packing are from H⋯H (56.9%), O⋯H/H⋯O (23.0%) and C⋯H/H⋯C (19.6%) contacts.

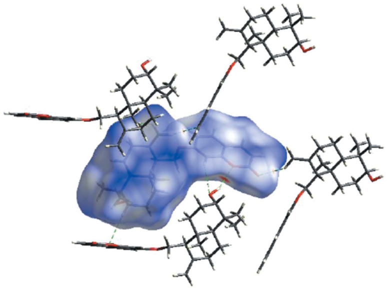

### 1. Chemical context

The genus *Ferula* belongs to the *Apiaceae* family, which comprises over 160 species and is spread throughout the world, including the Mediterranean region, Central Asia, Siberia, China, Afghanistan, Iran, North Africa and the Caucasus (Mir-Babayev & Houghton, 2002; Çelik *et al.*, 2023). This genus is rich in sesquiterpene coumarins. Extracts of *Ferula* species exhibit antimicrobial and estrogenic effects, and are natural plant-growth inhibitors and stimulants. Therefore, they have long been well known in popular medicine for the treatment of various health disorders, such as cough, asthma, toothache and gastroenteric problems (Salehi *et al.*, 2019; Tapera *et al.*, 2022; Lakhrissi *et al.*, 2022). These plants have been used for oleo-gum resin, plant extracts and essential oils. The extracts and essential oils of different species of *Ferula* can also be used as natural food preservatives due to their antioxidant and antimicrobial activity (Daneshniya *et al.*, 2021; Chalkha *et al.*, 2023).

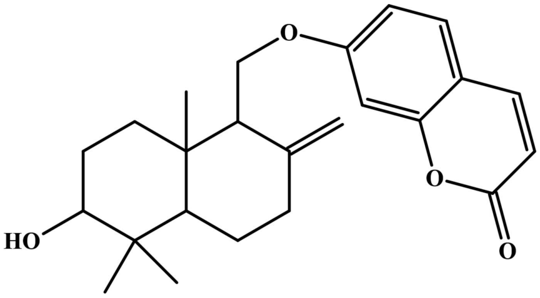

OPEN ACCESS
Published under a CC BY 4.0 licence

**Table 1**
Hydrogen-bond geometry (Å, °).

| $D$—H$\cdots$$A$ | $D$—H | H$\cdots$$A$ | $D$$\cdots$$A$ | $D$—H$\cdots$$A$ |
|---|---|---|---|---|
| O16—H16O$\cdots$O1$^{i}$ | 0.91 (7) | 2.34 (7) | 3.194 (4) | 158 (6) |
| C6—H6$\cdots$O2$^{ii}$ | 0.95 | 2.54 | 3.355 (6) | 144 |
| C8—H8$\cdots$O16$^{iii}$ | 0.95 | 2.59 | 3.256 (5) | 127 |
| C16—H16$\cdots$$Cg2^{iv}$ | 1.02 (5) | 2.69 (5) | 3.564 (5) | 146 (4) |
| C21—H21$A$$\cdots$$Cg1^{v}$ | 0.98 | 2.96 | 3.923 (6) | 169 |

Symmetry codes: (i) $x+\frac{1}{2}, -y+\frac{1}{2}, -z+1$; (ii) $-x+1, y+\frac{1}{2}, -z+\frac{3}{2}$; (iii) $x-\frac{1}{2}, -y+\frac{1}{2}, -z+1$; (iv) $-x-1, y+\frac{1}{2}, -z+\frac{3}{2}$; (v) $-x+\frac{1}{2}, -y+1, z-\frac{1}{2}$.

**Table 2**
Summary of short interatomic contacts (Å) in the title compound.

| Atom pair | Distance | Symmetry code |
|---|---|---|
| O2$\cdots$H4 | 2.66 | $x-1, y, z$ |
| O1$\cdots$H16O | 2.34 | $x-\frac{1}{2}, -y+\frac{1}{2}, -z+1$ |
| H4$\cdots$H20$B$ | 2.51 | $-x+1, y-\frac{1}{2}, -z+\frac{3}{2}$ |
| O2$\cdots$H20$B$ | 2.63 | $-x, y-\frac{1}{2}, -z+\frac{3}{2}$ |
| C4$A$$\cdots$H21$A$ | 2.91 | $-x+\frac{1}{2}, -y+1, z+\frac{1}{2}$ |
| H12$A$$\cdots$H20$A$ | 2.28 | $x+\frac{1}{2}, -y+\frac{3}{2}, -z+1$ |

Herein, we report the molecular and crystal structure, and Hirshfeld surface analysis of the title compound, 7-[(6-hydroxy-5,5,8a-trimethyl-2-methylenedecahydronaphthalen-1-yl)methoxy]-2*H*-chromen-2-one, extracted from *Ferula persica*.

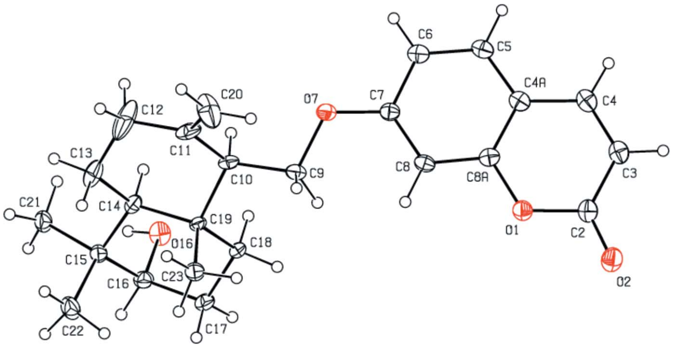

**Figure 1**
The molecular structure of the title compound, showing the atom labelling and displacement ellipsoids drawn at the 30% probability level.

### 2. Structural commentary

The molecular structure of the title compound (Fig. 1) consists of two *trans*-fused cyclohexane rings (atoms C10–C14/C19 and C14–C19) attached to a chromen-2-one moiety through an oxymethylene bridge. The cyclohexane rings adopt half-chair and chair conformations, respectively. The puckering parameters (Cremer & Pople, 1975) are $Q_T$ = 0.550 (5) Å, $\theta$ = 14.2 (5)° and $\varphi$ = 292 (2)° for C10–C14/C19, and $Q_T$ = 0.546 (5) Å, $\theta$ = 5.1 (5)° and $\varphi$ = 199 (5)° for C14–C19. The bond lengths and angles within the title molecule are in agreement with those reported for similar compounds compiled in the *Database survey* section.

### 3. Supramolecular features and Hirshfeld surface analysis

In the crystal of the title compound, molecules are linked by C—H$\cdots$O and O—H$\cdots$O hydrogen bonds, forming a triperiodic network (Tables 1 and 2, and Figs. 2–4). Furthermore, C—H$\cdots$$\pi$ interactions consolidate the crystal structure (Table 1 and Figs. 5 and 6).

Hirshfeld surfaces and two-dimensional (2D) fingerprint plots were produced using *CrystalExplorer17.5* (Spackman *et al.*, 2021) to quantify the intermolecular interactions in the crystal. The Hirshfeld surface mapped over $d_{norm}$ in the range

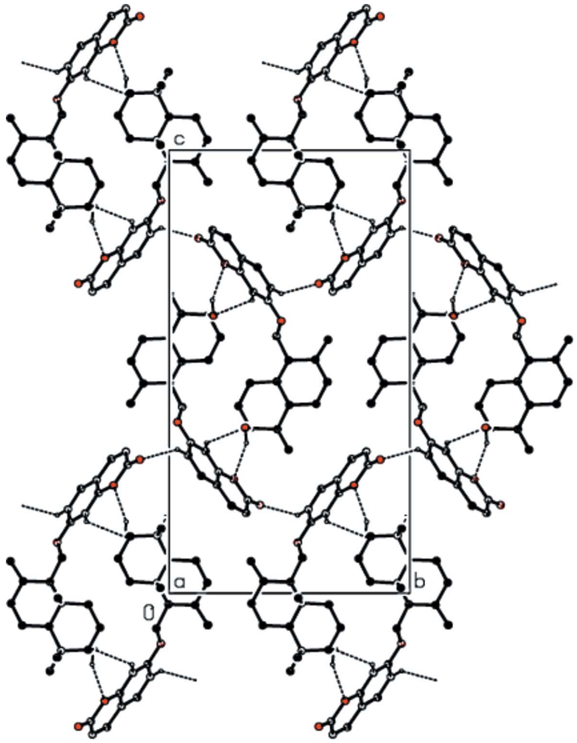

**Figure 2**
The crystal structure of the title compound in a view along [100]. O—H$\cdots$O and C—H$\cdots$O hydrogen bonds are indicated by dashed lines. H atoms not involved in hydrogen bonding have been omitted for clarity.

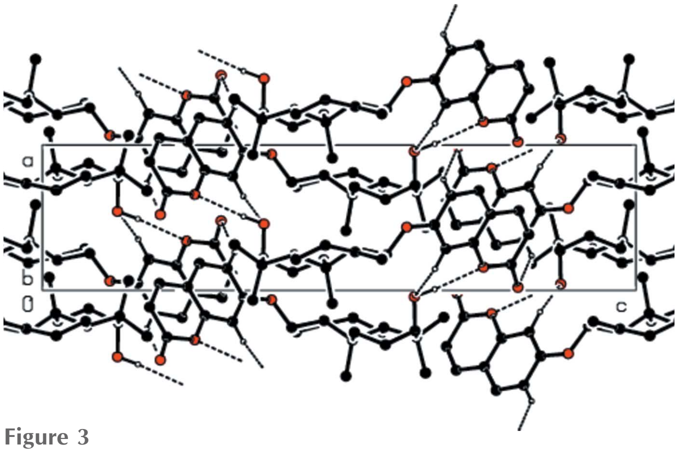

**Figure 3**
The crystal structure of the title compound in a view along [010]. O—H$\cdots$O and C—H$\cdots$O hydrogen bonds are indicated by dashed lines. H atoms not involved in hydrogen bonding have been omitted for clarity.

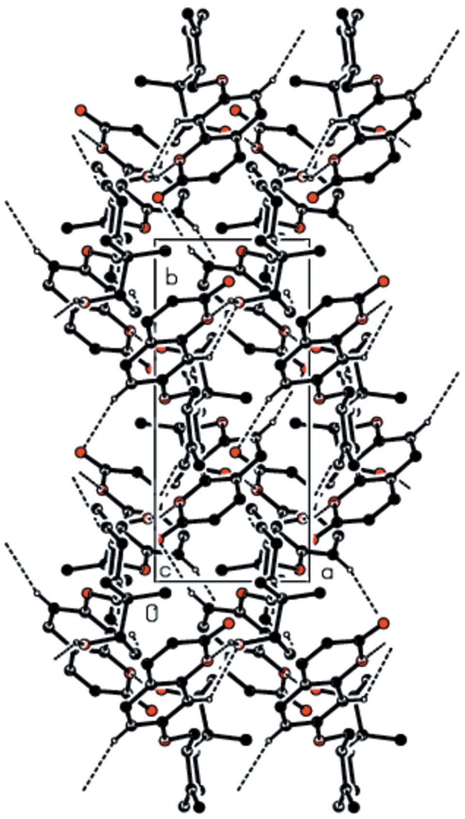

**Figure 4**
The crystal structure of the title compound in a view along [001]. O—H···O and C—H···O hydrogen bonds are indicated by dashed lines. H atoms not involved in hydrogen bonding have been omitted for clarity.

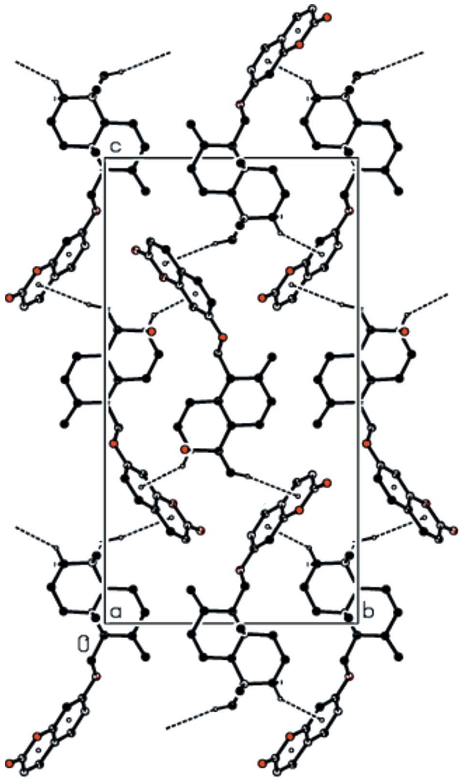

**Figure 5**
View along [100] of the C—H···$\pi$ interactions (dashed lines) in the crystal of the title compound.

from −0.2594 to +1.5213 a.u. is shown in Fig. 7, where significant intermolecular contacts appear as red-coloured spots, which indicate the O—H···O and C—H···O hydrogen-bonding interactions. The 2D fingerprint plots are presented in Fig. 8. The H···H contacts comprise 56.9% of the total interactions. The O···H/H···O (23.0%) and C···H/H···C (19.6%) interactions also make significant contributions to the total Hirshfeld surface. The C···C and O···C/C···O contacts contribute 0.2% of the total interactions.

### 4. Database survey

Six compounds similar to the 1,2,3,4,4a,5,8,8a-octahydronaphthalene group were found from a search of the Cambridge Structural Database (updated 20 March 2023; Groom *et al.*, 2016): 7-[(6-hydroxy-2,5,5,8a-tetramethyl-1,4,4a,5,6,7,8,8a-octahydronaphthalen-1-yl)methoxy]-2*H*-chromen-2-one, (**I**) (Bashir *et al.*, 2014); (*E*)-3-[(1*R**,2*S**,4a*S**,8a*R**)-2- (benzo[*d*][1,3]dioxol-5-yl)-1,2,4a,5,6,7,8,8a-octahydronaphthalen-1-yl]-*N*-isobutylacrylamide, (**II**) (Lenta *et al.*, 2015); 3-(4-bromophenyl)-l-[4-(4-bromophenyl)-3-butene-2-one-1-yl]-2-[3-(2,6,6-trimethyl-1-cyclohexen-l-yl)-2-propene-1-one-1-yl]-l,2,3,4,5,6,7,8-octahydro-8,8-dimethylnaphtalene, (**III**) (Ginderow, 1996); (+/−)3$\beta$-bromo-2$\beta$-methyl-4a$\beta H$,8a$\alpha H$-decahydronaphthalen-2$\alpha$-ol, (**IV**) (Fallon *et al.*, 1992); (2*R*,3*R*,5*R*)-2-[(2*R*,3a*S*,6a*R*)-2,3,3a,4,5,6a-hexahydrofuro[2,3-*b*]furan-2-yl]-5-isopropenyl-2,3-dimethylcyclohexanone, (**V**) (Ellis & Spek, 2000); (4a*R*,5*S*,7*R*)-5-isopropenyl-7,8,8-trimethyl-2,3,4,4a,5,6,7,8-octahydronaphthalene-4a-carbonitrile, (**VI**) (Ellis & Spek, 2000).

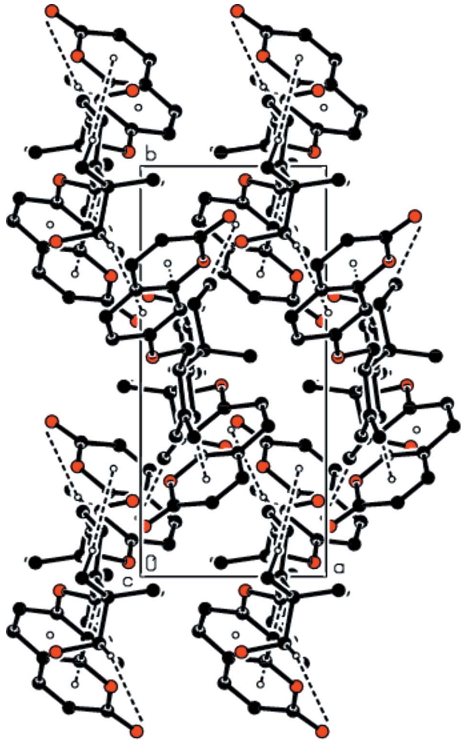

**Figure 6**
View along [001] of the C—H···$\pi$ interactions (dashed lines) in the crystal of the title compound.

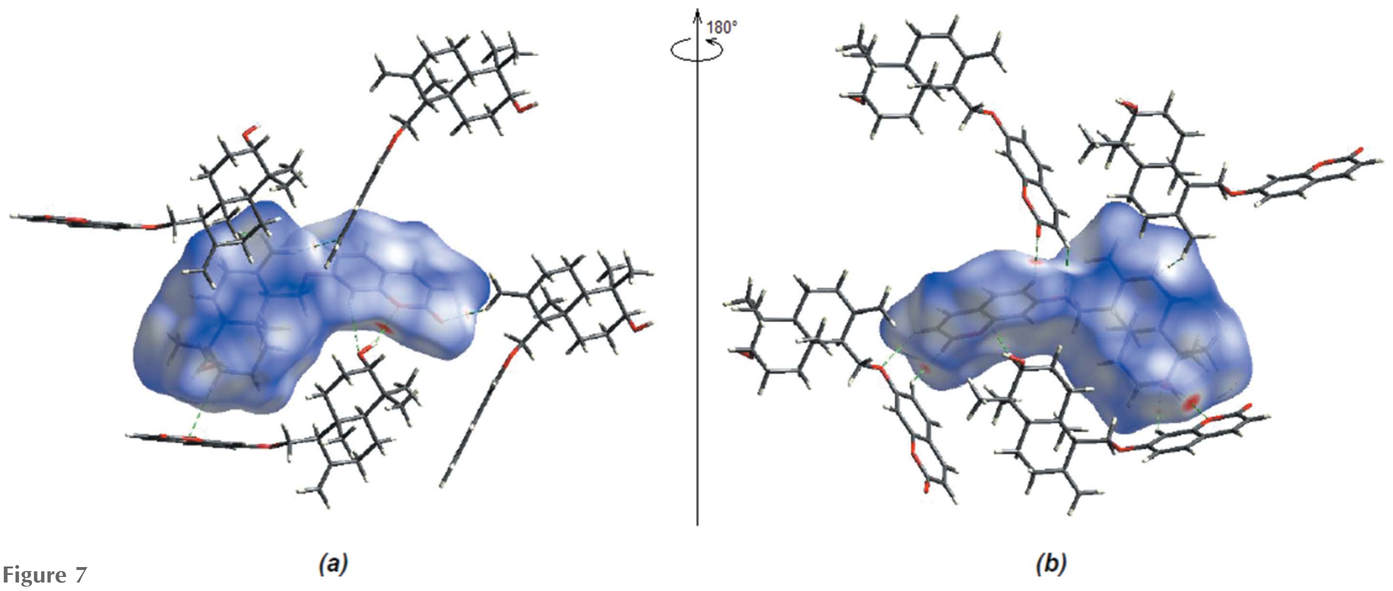

**Figure 7**
The (*a*) front and (*b*) back sides of the three-dimensional Hirshfeld surface of the title compound mapped over $d_{norm}$, with a fixed colour scale from −0.2594 to +1.5213 a.u.

In the crystal of (**I**), the molecules are connected by C—H···O and O—H···O hydrogen bonds, forming bands along [100] that are oriented parallel to (100). Additional C—H···π and van der Waals interactions result in a triperiodic network. In the crystal of (**II**), molecules are linked by N—H···O hydrogen bonds, forming chains propagating along [100]. The chains are linked by pairs of C—H···O hydrogen bonds, involving inversion-related benzodioxole ring systems, thus forming ribbons lying parallel to (010). There are also C—H···π interactions present within the ribbons. Disorder in the crystal of (**III**) arises from the co-existence of the two possible puckered conformations of the dimethylcyclohexene rings. In (**IV**), the *trans*-fused six-membered rings are chair-shaped but somewhat flattened. (**V**) and (**VI**) comprise the molecular structures of two chiral cyclohexanones based on *R*-(−)-carvone. The six-membered ring in (**V**) is in a chair conformation with the two fused five-membered rings of the furofuranyl substituent in a *cis* configuration. Compound (**VI**) contains a decalin group; one ring has the chair form, whilst the other is in a half-boat conformation.

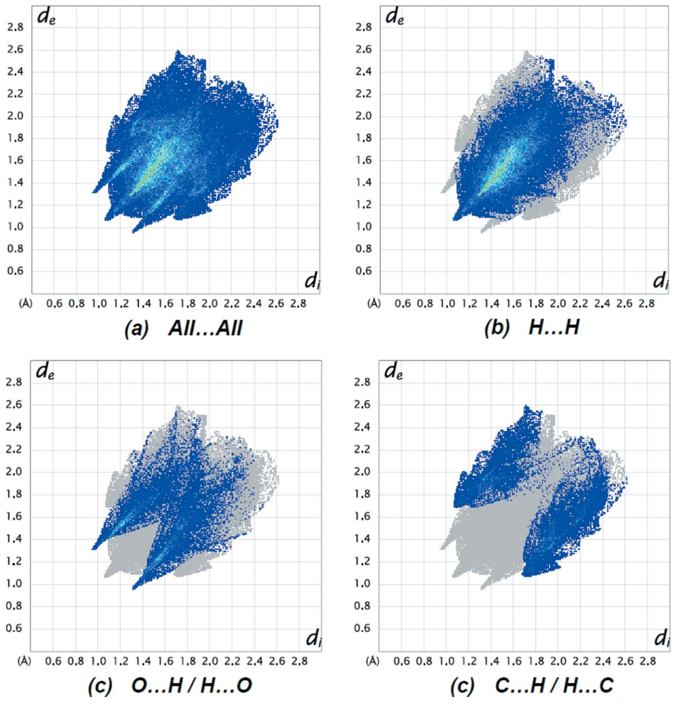

**Figure 8**
The 2D fingerprint plots of the title compound, showing (*a*) all interactions, and delineated into (*b*) H···H, (*c*) O···H/H···O and (*d*) C···H/H···C interactions. $d_e$ and $d_i$ represent the distances from a point on the Hirshfeld surface to the nearest atoms outside (external) and inside (internal) the surface, respectively.

### 5. Synthesis and crystallization

The title compound was isolated previously from *Ferula lehmanni* and fully characterized (Sagitdinova *et al.*, 1983). The present material was isolated from the roots of the *Ferula persica* herb by a similar method.

### 6. Refinement

Crystal data, data collection and structure refinement details are summarized in Table 3. The H atoms of the –OH group and the methine H atom attached to C16 were located in a difference map, and refined freely [O16—H16O = 0.90 (6) Å and C16—H16 = 1.02 (5) Å]. The remaining H atoms bound to C atoms were positioned geometrically and refined as riding, with C—H = 0.95 (aromatic), 0.99 (methylene), 0.98 (methyl) and 1.00 Å (methine), with $U_{iso}(H) = 1.5U_{eq}(C)$ for methyl H atoms and $1.2U_{eq}(C)$ for all others. Two reflections (590 and 2,15,0), for which *I*(obs) and *I*(calc) differed by more than 10σ, were removed from the refinement.

**Table 3**
Experimental details.

| | |
|---|---|
| Crystal data | |
| Chemical formula | $C_{24}H_{30}O_4$ |
| $M_r$ | 382.48 |
| Crystal system, space group | Orthorhombic, $P2_12_12_1$ |
| Temperature (K) | 100 |
| *a*, *b*, *c* (Å) | 6.0526 (1), 13.3964 (4), 24.6848 (5) |
| *V* (Å$^3$) | 2001.52 (8) |
| *Z* | 4 |
| Radiation type | Cu *K*α |
| μ (mm$^{-1}$) | 0.68 |
| Crystal size (mm) | 0.20 × 0.14 × 0.09 |
| | |
| Data collection | |
| Diffractometer | Rigaku XtaLAB Synergy Dualflex diffractometer with a HyPix detector |
| Absorption correction | Multi-scan (*CrysAlis PRO*; Rigaku OD, 2022) |
| $T_{min}$, $T_{max}$ | 0.664, 1.000 |
| No. of measured, independent and observed [$I > 2\sigma(I)$] reflections | 11367, 3918, 3687 |
| $R_{int}$ | 0.034 |
| $(\sin \theta/\lambda)_{max}$ (Å$^{-1}$) | 0.633 |
| | |
| Refinement | |
| $R[F^2 > 2\sigma(F^2)]$, $wR(F^2)$, $S$ | 0.062, 0.158, 1.12 |
| No. of reflections | 3918 |
| No. of parameters | 264 |
| H-atom treatment | H atoms treated by a mixture of independent and constrained refinement |
| $\Delta\rho_{max}$, $\Delta\rho_{min}$ (e Å$^{-3}$) | 0.43, −0.56 |
| Absolute structure | Flack *x* determined using 1307 quotients $[(I^+) - (I^-)]/[(I^+) + (I^-)]$ (Parsons *et al.*, 2013) |
| Absolute structure parameter | −0.18 (12) |

Computer programs: *CrysAlis PRO* (Rigaku OD, 2022), *SHELXT* (Sheldrick, 2015*a*), *SHELXL2018* (Sheldrick, 2015*b*), *ORTEP-3 for Windows* (Farrugia, 2012) and *PLATON* (Spek, 2020).

### Acknowledgements

This study was supported by Baku State University, Azerbaijan Medical University and the RUDN University Strategic Academic Leadership Program. Authors contributions are as follows: conceptualization, ANK and IGM; methodology, ANK, EGK and IGM; investigation, ANK, MA and EGK; writing (original draft), MA and ANK; writing (review and editing of the manuscript), MA and ANK; visualization, MA, ANK and IGM; funding acquisition, VNK, AB and ANK; resources, AB, VNK and ANA; supervision, ANK and MA.

### References

Bashir, S., Alam, M., Adhikari, A., Shrestha, R. L. S., Yousuf, S., Ahmad, B., Parveen, S., Aman, A. & Iqbal Choudhary, M. (2014). *Phytochem. Lett.* **9**, 46–50.
Çelik, M. S., Çetinus, A., Yenidünya, A. F., Çetinkaya, S. & Tüzün, B. (2023). *J. Mol. Struct.* **1272**, 134158.
Chalkha, M., Ameziane el Hassani, A., Nakkabi, A., Tüzün, B., Bakhouch, M., Benjelloun, A. T., Sfaira, M., Saadi, M., Ammari, L. E. & Yazidi, M. E. (2023). *J. Mol. Struct.* **1273**, 134255.
Cremer, D. & Pople, J. A. (1975). *J. Am. Chem. Soc.* **97**, 1354–1358.
Daneshniya, M., Maleki, M. H., Mohammadi, M. A., Ahangarian, K., Kondeskalaei, V. J. & Alavi, H. (2021). *SARJNP*, **4**, 1–23.
Ellis, D. D. & Spek, A. L. (2000). *Acta Cryst.* C**56**, 1173–1175.
Fallon, G. D., Gatehouse, B. M., Middleton, S. & Vanni, S. P. (1992). *Acta Cryst.* B**48**, 227–230.
Farrugia, L. J. (2012). *J. Appl. Cryst.* **45**, 849–854.
Ginderow, D. (1996). *Acta Cryst.* C**52**, 256–259.
Groom, C. R., Bruno, I. J., Lightfoot, M. P. & Ward, S. C. (2016). *Acta Cryst.* B**72**, 171–179.
Lakhrissi, Y., Rbaa, M., Tuzun, B., Hichar, A., Anouar, H., Ounine, K., Almalki, F., Hadda, T. B., Zarrouk, A. & Lakhrissi, B. (2022). *J. Mol. Struct.* **1259**, 132683.
Lenta, B. N., Chouna, R. J., Neumann, B., Stammler, H.-G. & Sewald, N. (2015). *Acta Cryst.* E**71**, o457–o458.
Mir-Babayev, N. F. & Houghton, P. J. (2002). *Pharm. Biol.* **40**, 16–22.
Parsons, S., Flack, H. D. & Wagner, T. (2013). *Acta Cryst.* B**69**, 249–259.
Rigaku OD (2022). *CrysAlis PRO*. Rigaku Oxford Diffraction, Yarnton, Oxfordshire, England.
Sagitdinova, G. V., Saidkhodzhaev, A. I. & Malikov, V. M. (1983). *Khim. Prir. Soedin.* **6**, 709–712.
Salehi, M., Naghavi, M. R. & Bahmankar, M. (2019). *Ind. Crops Prod.* **139**, 111511.
Sheldrick, G. M. (2015*a*). *Acta Cryst.* A**71**, 3–8.
Sheldrick, G. M. (2015*b*). *Acta Cryst.* C**71**, 3–8.
Spackman, P. R., Turner, M. J., McKinnon, J. J., Wolff, S. K., Grimwood, D. J., Jayatilaka, D. & Spackman, M. A. (2021). *J. Appl. Cryst.* **54**, 1006–1011.
Spek, A. L. (2020). *Acta Cryst.* E**76**, 1–11.
Tapera, M., Kekeçmuhammed, H., Tüzün, B., Sarıpınar, E., Koçyiğit, M., Yıldırım, E., Doğan, M. & Zorlu, Y. (2022). *J. Mol. Struct.* **1269**, 133816.

### supporting information

*Acta Cryst.* (2023). E**79**, 777-781 [https://doi.org/10.1107/S2056989023006552]

### Crystal structure and Hirshfeld surface analysis of 7-[(6-hydroxy-5,5,8a-trimethyl-2-methylenedecahydronaphthalen-1-yl)methoxy]-2*H*-chromen-2-one

**Elvin G. Karimli, Victor N. Khrustalev, Mehmet Akkurt, Ali N. Khalilov, Ajaya Bhattarai, Adila N. Aleskerova and İbrahim G. Mamedov**

### Computing details

Data collection: *CrysAlis PRO* (Rigaku OD, 2022); cell refinement: *CrysAlis PRO* (Rigaku OD, 2022); data reduction: *CrysAlis PRO* (Rigaku OD, 2022); program(s) used to solve structure: SHELXT (Sheldrick, 2015a); program(s) used to refine structure: *SHELXL2018* (Sheldrick, 2015b); molecular graphics: *ORTEP-3 for Windows* (Farrugia, 2012); software used to prepare material for publication: *PLATON* (Spek, 2020).

### 7-[(6-Hydroxy-5,5,8a-trimethyl-2-methylenedecahydronaphthalen-1-yl)methoxy]-2*H*-chromen-2-one

*Crystal data*

$C_{24}H_{30}O_4$
$M_r$ = 382.48
Orthorhombic, $P2_12_12_1$
$a$ = 6.0526 (1) Å
$b$ = 13.3964 (4) Å
$c$ = 24.6848 (5) Å
$V$ = 2001.52 (8) Å$^3$
$Z$ = 4
$F$(000) = 824
$D_x$ = 1.269 Mg m$^{-3}$
Cu $K\alpha$ radiation, $\lambda$ = 1.54184 Å
Cell parameters from 7116 reflections
$\theta$ = 3.7–77.5°
$\mu$ = 0.68 mm$^{-1}$
$T$ = 100 K
Prism, colourless
0.20 × 0.14 × 0.09 mm

*Data collection*

Rigaku XtaLAB Synergy Dualflex diffractometer with a HyPix detector
Radiation source: micro-focus sealed X-ray tube
$\varphi$ and $\omega$ scans
Absorption correction: multi-scan (CrysAlis PRO; Rigaku OD, 2022)
$T_{min}$ = 0.664, $T_{max}$ = 1.000
11367 measured reflections
3918 independent reflections
3687 reflections with $I > 2\sigma(I)$
$R_{int}$ = 0.034
$\theta_{max}$ = 77.5°, $\theta_{min}$ = 3.6°
$h = -7 \rightarrow 7$
$k = -16 \rightarrow 16$
$l = -25 \rightarrow 31$

*Refinement*

Refinement on $F^2$
Least-squares matrix: full
$R[F^2 > 2\sigma(F^2)]$ = 0.062
$wR(F^2)$ = 0.158
$S$ = 1.12
3918 reflections
264 parameters
0 restraints
Hydrogen site location: mixed
H atoms treated by a mixture of independent and constrained refinement
$w = 1/[\sigma^2(F_o^2) + (0.0424P)^2 + 2.9169P]$ where $P = (F_o^2 + 2F_c^2)/3$
$(\Delta/\sigma)_{max} < 0.001$
$\Delta\rho_{max}$ = 0.43 e Å$^{-3}$
$\Delta\rho_{min}$ = −0.56 e Å$^{-3}$

Absolute structure: Flack *x* determined using 1307 quotients [(I+)-(I-)]/[(I+)+(I-)] (Parsons *et al.*, 2013)
Absolute structure parameter: −0.18 (12)

*Special details*

**Geometry**. All esds (except the esd in the dihedral angle between two l.s. planes) are estimated using the full covariance matrix. The cell esds are taken into account individually in the estimation of esds in distances, angles and torsion angles; correlations between esds in cell parameters are only used when they are defined by crystal symmetry. An approximate (isotropic) treatment of cell esds is used for estimating esds involving l.s. planes.

*Fractional atomic coordinates and isotropic or equivalent isotropic displacement parameters (Å²)*

| | *x* | *y* | *z* | $U_{iso}$*/$U_{eq}$ |
|---|---|---|---|---|
| O1 | 0.1603 (5) | 0.2319 (2) | 0.74312 (12) | 0.0304 (7) |
| C2 | 0.1791 (8) | 0.1730 (4) | 0.78982 (18) | 0.0318 (10) |
| O2 | 0.0215 (6) | 0.1226 (3) | 0.80119 (13) | 0.0405 (8) |
| C3 | 0.3855 (8) | 0.1803 (4) | 0.81926 (18) | 0.0327 (10) |
| H3 | 0.405572 | 0.141392 | 0.851048 | 0.039* |
| C4 | 0.5488 (8) | 0.2406 (3) | 0.80275 (17) | 0.0319 (10) |
| H4 | 0.682740 | 0.243074 | 0.822775 | 0.038* |
| C4A | 0.5245 (7) | 0.3017 (3) | 0.75521 (17) | 0.0277 (9) |
| C5 | 0.6868 (7) | 0.3667 (3) | 0.73537 (18) | 0.0290 (9) |
| H5 | 0.822769 | 0.373281 | 0.754240 | 0.035* |
| C6 | 0.6516 (8) | 0.4212 (3) | 0.68878 (18) | 0.0301 (9) |
| H6 | 0.762016 | 0.465638 | 0.675843 | 0.036* |
| C7 | 0.4511 (7) | 0.4107 (3) | 0.66054 (17) | 0.0266 (9) |
| O7 | 0.4349 (5) | 0.4653 (2) | 0.61394 (11) | 0.0286 (7) |
| C8 | 0.2858 (7) | 0.3495 (3) | 0.67958 (17) | 0.0265 (9) |
| H8 | 0.147774 | 0.344923 | 0.661441 | 0.032* |
| C8A | 0.3277 (7) | 0.2944 (3) | 0.72632 (17) | 0.0267 (9) |
| C9 | 0.2455 (7) | 0.4463 (3) | 0.57989 (17) | 0.0277 (9) |
| H9A | 0.110286 | 0.474519 | 0.596468 | 0.033* |
| H9B | 0.224179 | 0.373572 | 0.574977 | 0.033* |
| C10 | 0.2921 (6) | 0.4964 (3) | 0.52545 (17) | 0.0250 (8) |
| H10 | 0.451638 | 0.483852 | 0.517389 | 0.030* |
| C11 | 0.2644 (8) | 0.6075 (3) | 0.5269 (2) | 0.0365 (11) |
| C12 | 0.3107 (17) | 0.6579 (4) | 0.4766 (2) | 0.080 (3) |
| H12A | 0.472930 | 0.661076 | 0.471769 | 0.096* |
| H12B | 0.255956 | 0.727365 | 0.479326 | 0.096* |
| C13 | 0.2122 (9) | 0.6113 (4) | 0.4270 (2) | 0.0433 (12) |
| H13A | 0.279824 | 0.641920 | 0.394525 | 0.052* |
| H13B | 0.051974 | 0.625974 | 0.426186 | 0.052* |
| C14 | 0.2453 (7) | 0.4992 (3) | 0.42470 (17) | 0.0261 (8) |
| H14 | 0.409159 | 0.490292 | 0.426388 | 0.031* |
| C15 | 0.1770 (7) | 0.4497 (4) | 0.36979 (16) | 0.0289 (9) |
| C16 | 0.2232 (7) | 0.3372 (4) | 0.37214 (18) | 0.0298 (9) |
| O16 | 0.4562 (5) | 0.3160 (3) | 0.37334 (13) | 0.0353 (7) |
| C17 | 0.1259 (8) | 0.2879 (3) | 0.42204 (17) | 0.0309 (9) |

| | | | | |
|---|---|---|---|---|
| H17A | 0.164982 | 0.216149 | 0.422058 | 0.037* |
| H17B | −0.037133 | 0.293255 | 0.420808 | 0.037* |
| C18 | 0.2109 (7) | 0.3362 (3) | 0.47414 (16) | 0.0240 (8) |
| H18A | 0.372883 | 0.326882 | 0.476324 | 0.029* |
| H18B | 0.143699 | 0.301914 | 0.505609 | 0.029* |
| C19 | 0.1583 (6) | 0.4484 (3) | 0.47759 (15) | 0.0209 (8) |
| C20 | 0.2133 (13) | 0.6603 (5) | 0.5719 (2) | 0.067 (2) |
| H20A | 0.207618 | 0.731071 | 0.570476 | 0.080* |
| H20B | 0.183005 | 0.626517 | 0.604933 | 0.080* |
| C21 | 0.3154 (8) | 0.4964 (4) | 0.32367 (17) | 0.0347 (10) |
| H21A | 0.259824 | 0.563489 | 0.315693 | 0.052* |
| H21B | 0.303667 | 0.454693 | 0.291171 | 0.052* |
| H21C | 0.470411 | 0.500524 | 0.334907 | 0.052* |
| C22 | −0.0672 (8) | 0.4665 (5) | 0.3545 (2) | 0.0424 (13) |
| H22A | −0.162189 | 0.430585 | 0.380056 | 0.064* |
| H22B | −0.093601 | 0.441732 | 0.317737 | 0.064* |
| H22C | −0.100894 | 0.538043 | 0.356051 | 0.064* |
| C23 | −0.0900 (7) | 0.4630 (4) | 0.48846 (17) | 0.0300 (10) |
| H23A | −0.175588 | 0.428509 | 0.460426 | 0.045* |
| H23B | −0.125252 | 0.534374 | 0.487810 | 0.045* |
| H23C | −0.127311 | 0.435337 | 0.524059 | 0.045* |
| H16 | 0.162 (9) | 0.307 (4) | 0.338 (2) | 0.031 (13)* |
| H16O | 0.508 (11) | 0.321 (5) | 0.339 (3) | 0.054 (17)* |

*Atomic displacement parameters (Å$^2$)*

| | $U^{11}$ | $U^{22}$ | $U^{33}$ | $U^{12}$ | $U^{13}$ | $U^{23}$ |
|---|---|---|---|---|---|---|
| O1 | 0.0258 (14) | 0.0338 (17) | 0.0317 (15) | 0.0023 (14) | 0.0012 (13) | 0.0019 (13) |
| C2 | 0.033 (2) | 0.030 (2) | 0.032 (2) | 0.008 (2) | 0.0034 (19) | 0.0009 (17) |
| O2 | 0.0323 (18) | 0.045 (2) | 0.0446 (18) | 0.0045 (17) | 0.0059 (15) | 0.0103 (15) |
| C3 | 0.038 (2) | 0.028 (2) | 0.032 (2) | 0.009 (2) | −0.0008 (18) | −0.0023 (18) |
| C4 | 0.037 (2) | 0.027 (2) | 0.032 (2) | 0.011 (2) | −0.004 (2) | −0.0058 (17) |
| C4A | 0.028 (2) | 0.0245 (19) | 0.030 (2) | 0.0069 (18) | −0.0001 (17) | −0.0082 (16) |
| C5 | 0.025 (2) | 0.024 (2) | 0.038 (2) | 0.0066 (18) | −0.0055 (18) | −0.0080 (18) |
| C6 | 0.026 (2) | 0.027 (2) | 0.037 (2) | 0.0019 (19) | −0.0011 (18) | −0.0053 (17) |
| C7 | 0.026 (2) | 0.025 (2) | 0.0289 (19) | 0.0002 (18) | −0.0003 (17) | −0.0042 (16) |
| O7 | 0.0243 (14) | 0.0292 (16) | 0.0324 (15) | −0.0032 (13) | −0.0040 (12) | 0.0000 (12) |
| C8 | 0.0198 (19) | 0.030 (2) | 0.030 (2) | 0.0021 (18) | −0.0015 (16) | −0.0048 (17) |
| C8A | 0.0255 (19) | 0.023 (2) | 0.0311 (19) | 0.0017 (18) | 0.0023 (17) | −0.0055 (16) |
| C9 | 0.0219 (18) | 0.032 (2) | 0.0296 (19) | −0.0049 (18) | −0.0031 (16) | −0.0035 (18) |
| C10 | 0.0158 (17) | 0.024 (2) | 0.035 (2) | −0.0048 (16) | 0.0009 (16) | −0.0031 (17) |
| C11 | 0.030 (2) | 0.020 (2) | 0.060 (3) | −0.0065 (19) | −0.001 (2) | −0.005 (2) |
| C12 | 0.169 (9) | 0.020 (2) | 0.051 (3) | −0.009 (4) | 0.036 (5) | 0.001 (2) |
| C13 | 0.052 (3) | 0.027 (2) | 0.051 (3) | 0.006 (2) | 0.013 (3) | 0.015 (2) |
| C14 | 0.0222 (18) | 0.023 (2) | 0.033 (2) | 0.0005 (17) | 0.0040 (17) | 0.0071 (17) |
| C15 | 0.0196 (17) | 0.038 (2) | 0.029 (2) | −0.0034 (19) | −0.0017 (16) | 0.0098 (19) |
| C16 | 0.031 (2) | 0.031 (2) | 0.027 (2) | −0.0076 (19) | −0.0012 (18) | −0.0027 (17) |
| O16 | 0.0343 (16) | 0.0376 (18) | 0.0341 (16) | 0.0092 (15) | 0.0053 (15) | −0.0005 (14) |

| | | | | | | |
|---|---|---|---|---|---|---|
| C17 | 0.036 (2) | 0.024 (2) | 0.032 (2) | −0.0095 (19) | −0.0024 (19) | −0.0003 (18) |
| C18 | 0.028 (2) | 0.0175 (19) | 0.0271 (19) | −0.0058 (17) | −0.0028 (17) | 0.0025 (15) |
| C19 | 0.0153 (16) | 0.0184 (19) | 0.0289 (18) | −0.0030 (16) | −0.0006 (15) | 0.0033 (16) |
| C20 | 0.118 (6) | 0.040 (3) | 0.042 (3) | 0.021 (4) | −0.024 (4) | −0.011 (2) |
| C21 | 0.027 (2) | 0.047 (3) | 0.030 (2) | 0.000 (2) | 0.0008 (18) | 0.013 (2) |
| C22 | 0.025 (2) | 0.065 (4) | 0.038 (2) | 0.001 (2) | −0.0041 (19) | 0.018 (2) |
| C23 | 0.0196 (19) | 0.038 (3) | 0.032 (2) | −0.0009 (19) | 0.0011 (16) | 0.0049 (19) |

*Geometric parameters (Å, º)*

| | | | |
|---|---|---|---|
| O1—C8A | 1.378 (5) | C13—H13A | 0.9900 |
| O1—C2 | 1.401 (5) | C13—H13B | 0.9900 |
| C2—O2 | 1.202 (6) | C14—C19 | 1.563 (5) |
| C2—C3 | 1.449 (7) | C14—C15 | 1.565 (6) |
| C3—C4 | 1.340 (7) | C14—H14 | 1.0000 |
| C3—H3 | 0.9500 | C15—C16 | 1.534 (7) |
| C4—C4A | 1.438 (6) | C15—C22 | 1.542 (6) |
| C4—H4 | 0.9500 | C15—C21 | 1.545 (6) |
| C4A—C8A | 1.392 (6) | C16—O16 | 1.439 (5) |
| C4A—C5 | 1.401 (7) | C16—C17 | 1.516 (6) |
| C5—C6 | 1.379 (6) | C16—H16 | 1.02 (5) |
| C5—H5 | 0.9500 | O16—H16O | 0.90 (6) |
| C6—C7 | 1.407 (6) | C17—C18 | 1.529 (6) |
| C6—H6 | 0.9500 | C17—H17A | 0.9900 |
| C7—O7 | 1.367 (5) | C17—H17B | 0.9900 |
| C7—C8 | 1.376 (6) | C18—C19 | 1.539 (6) |
| O7—C9 | 1.444 (5) | C18—H18A | 0.9900 |
| C8—C8A | 1.393 (6) | C18—H18B | 0.9900 |
| C8—H8 | 0.9500 | C19—C23 | 1.539 (5) |
| C9—C10 | 1.528 (6) | C20—H20A | 0.9500 |
| C9—H9A | 0.9900 | C20—H20B | 0.9500 |
| C9—H9B | 0.9900 | C21—H21A | 0.9800 |
| C10—C11 | 1.498 (6) | C21—H21B | 0.9800 |
| C10—C19 | 1.570 (5) | C21—H21C | 0.9800 |
| C10—H10 | 1.0000 | C22—H22A | 0.9800 |
| C11—C20 | 1.352 (8) | C22—H22B | 0.9800 |
| C11—C12 | 1.443 (8) | C22—H22C | 0.9800 |
| C12—C13 | 1.496 (9) | C23—H23A | 0.9800 |
| C12—H12A | 0.9900 | C23—H23B | 0.9800 |
| C12—H12B | 0.9900 | C23—H23C | 0.9800 |
| C13—C14 | 1.516 (6) | | |
| | | | |
| C8A—O1—C2 | 122.0 (4) | C13—C14—C15 | 114.7 (4) |
| O2—C2—O1 | 116.4 (4) | C19—C14—C15 | 116.8 (3) |
| O2—C2—C3 | 127.2 (4) | C13—C14—H14 | 104.3 |
| O1—C2—C3 | 116.4 (4) | C19—C14—H14 | 104.3 |
| C4—C3—C2 | 121.6 (4) | C15—C14—H14 | 104.3 |
| C4—C3—H3 | 119.2 | C16—C15—C22 | 109.1 (4) |

| | |
|---|---|
| C2—C3—H3 | 119.2 |
| C3—C4—C4A | 121.1 (4) |
| C3—C4—H4 | 119.5 |
| C4A—C4—H4 | 119.5 |
| C8A—C4A—C5 | 117.7 (4) |
| C8A—C4A—C4 | 117.7 (4) |
| C5—C4A—C4 | 124.6 (4) |
| C6—C5—C4A | 120.8 (4) |
| C6—C5—H5 | 119.6 |
| C4A—C5—H5 | 119.6 |
| C5—C6—C7 | 119.6 (4) |
| C5—C6—H6 | 120.2 |
| C7—C6—H6 | 120.2 |
| O7—C7—C8 | 123.7 (4) |
| O7—C7—C6 | 115.2 (4) |
| C8—C7—C6 | 121.2 (4) |
| C7—O7—C9 | 116.9 (3) |
| C7—C8—C8A | 117.8 (4) |
| C7—C8—H8 | 121.1 |
| C8A—C8—H8 | 121.1 |
| O1—C8A—C4A | 121.2 (4) |
| O1—C8A—C8 | 115.9 (4) |
| C4A—C8A—C8 | 122.9 (4) |
| O7—C9—C10 | 106.7 (3) |
| O7—C9—H9A | 110.4 |
| C10—C9—H9A | 110.4 |
| O7—C9—H9B | 110.4 |
| C10—C9—H9B | 110.4 |
| H9A—C9—H9B | 108.6 |
| C11—C10—C9 | 113.2 (4) |
| C11—C10—C19 | 111.5 (4) |
| C9—C10—C19 | 112.8 (3) |
| C11—C10—H10 | 106.2 |
| C9—C10—H10 | 106.2 |
| C19—C10—H10 | 106.2 |
| C20—C11—C12 | 120.5 (5) |
| C20—C11—C10 | 124.4 (5) |
| C12—C11—C10 | 115.0 (5) |
| C11—C12—C13 | 115.6 (5) |
| C11—C12—H12A | 108.4 |
| C13—C12—H12A | 108.4 |
| C11—C12—H12B | 108.4 |
| C13—C12—H12B | 108.4 |
| H12A—C12—H12B | 107.5 |
| C12—C13—C14 | 113.1 (5) |
| C12—C13—H13A | 109.0 |
| C14—C13—H13A | 109.0 |
| C12—C13—H13B | 109.0 |
| C16—C15—C21 | 109.1 (4) |
| C22—C15—C21 | 106.3 (3) |
| C16—C15—C14 | 109.6 (3) |
| C22—C15—C14 | 113.8 (4) |
| C21—C15—C14 | 108.9 (4) |
| O16—C16—C17 | 106.1 (4) |
| O16—C16—C15 | 111.9 (4) |
| C17—C16—C15 | 112.8 (4) |
| O16—C16—H16 | 107 (3) |
| C17—C16—H16 | 111 (3) |
| C15—C16—H16 | 107 (3) |
| C16—O16—H16O | 108 (4) |
| C16—C17—C18 | 111.6 (3) |
| C16—C17—H17A | 109.3 |
| C18—C17—H17A | 109.3 |
| C16—C17—H17B | 109.3 |
| C18—C17—H17B | 109.3 |
| H17A—C17—H17B | 108.0 |
| C17—C18—C19 | 113.0 (3) |
| C17—C18—H18A | 109.0 |
| C19—C18—H18A | 109.0 |
| C17—C18—H18B | 109.0 |
| C19—C18—H18B | 109.0 |
| H18A—C18—H18B | 107.8 |
| C18—C19—C23 | 109.6 (3) |
| C18—C19—C14 | 108.0 (3) |
| C23—C19—C14 | 114.8 (3) |
| C18—C19—C10 | 109.6 (3) |
| C23—C19—C10 | 108.7 (3) |
| C14—C19—C10 | 106.1 (3) |
| C11—C20—H20A | 120.0 |
| C11—C20—H20B | 120.0 |
| H20A—C20—H20B | 120.0 |
| C15—C21—H21A | 109.5 |
| C15—C21—H21B | 109.5 |
| H21A—C21—H21B | 109.5 |
| C15—C21—H21C | 109.5 |
| H21A—C21—H21C | 109.5 |
| H21B—C21—H21C | 109.5 |
| C15—C22—H22A | 109.5 |
| C15—C22—H22B | 109.5 |
| H22A—C22—H22B | 109.5 |
| C15—C22—H22C | 109.5 |
| H22A—C22—H22C | 109.5 |
| H22B—C22—H22C | 109.5 |
| C19—C23—H23A | 109.5 |
| C19—C23—H23B | 109.5 |
| H23A—C23—H23B | 109.5 |

| | | | |
|---|---|---|---|
| C14—C13—H13B | 109.0 | C19—C23—H23C | 109.5 |
| H13A—C13—H13B | 107.8 | H23A—C23—H23C | 109.5 |
| C13—C14—C19 | 110.8 (4) | H23B—C23—H23C | 109.5 |

| | | | |
|---|---|---|---|
| C8A—O1—C2—O2 | 178.7 (4) | C11—C12—C13—C14 | −46.2 (9) |
| C8A—O1—C2—C3 | −0.8 (6) | C12—C13—C14—C19 | 54.0 (6) |
| O2—C2—C3—C4 | −179.1 (5) | C12—C13—C14—C15 | −171.2 (5) |
| O1—C2—C3—C4 | 0.4 (6) | C13—C14—C15—C16 | 178.7 (4) |
| C2—C3—C4—C4A | 0.7 (7) | C19—C14—C15—C16 | −49.3 (4) |
| C3—C4—C4A—C8A | −1.4 (6) | C13—C14—C15—C22 | −58.8 (5) |
| C3—C4—C4A—C5 | 179.9 (4) | C19—C14—C15—C22 | 73.2 (5) |
| C8A—C4A—C5—C6 | 0.0 (6) | C13—C14—C15—C21 | 59.5 (5) |
| C4—C4A—C5—C6 | 178.7 (4) | C19—C14—C15—C21 | −168.5 (4) |
| C4A—C5—C6—C7 | −0.7 (6) | C22—C15—C16—O16 | 166.2 (4) |
| C5—C6—C7—O7 | −177.9 (4) | C21—C15—C16—O16 | 50.5 (5) |
| C5—C6—C7—C8 | 2.3 (6) | C14—C15—C16—O16 | −68.7 (4) |
| C8—C7—O7—C9 | −8.5 (6) | C22—C15—C16—C17 | −74.2 (4) |
| C6—C7—O7—C9 | 171.7 (4) | C21—C15—C16—C17 | 170.1 (3) |
| O7—C7—C8—C8A | 177.1 (4) | C14—C15—C16—C17 | 50.9 (5) |
| C6—C7—C8—C8A | −3.1 (6) | O16—C16—C17—C18 | 65.8 (5) |
| C2—O1—C8A—C4A | 0.1 (6) | C15—C16—C17—C18 | −57.1 (5) |
| C2—O1—C8A—C8 | −179.2 (4) | C16—C17—C18—C19 | 58.7 (5) |
| C5—C4A—C8A—O1 | 179.8 (4) | C17—C18—C19—C23 | 72.6 (4) |
| C4—C4A—C8A—O1 | 1.0 (6) | C17—C18—C19—C14 | −53.1 (4) |
| C5—C4A—C8A—C8 | −0.9 (6) | C17—C18—C19—C10 | −168.2 (3) |
| C4—C4A—C8A—C8 | −179.7 (4) | C13—C14—C19—C18 | −176.3 (4) |
| C7—C8—C8A—O1 | −178.2 (4) | C15—C14—C19—C18 | 49.9 (4) |
| C7—C8—C8A—C4A | 2.5 (6) | C13—C14—C19—C23 | 61.2 (5) |
| C7—O7—C9—C10 | −166.8 (3) | C15—C14—C19—C23 | −72.6 (5) |
| O7—C9—C10—C11 | −75.9 (4) | C13—C14—C19—C10 | −58.9 (4) |
| O7—C9—C10—C19 | 156.3 (3) | C15—C14—C19—C10 | 167.3 (3) |
| C9—C10—C11—C20 | 2.8 (7) | C11—C10—C19—C18 | 174.2 (4) |
| C19—C10—C11—C20 | 131.3 (6) | C9—C10—C19—C18 | −57.1 (4) |
| C9—C10—C11—C12 | 179.0 (5) | C11—C10—C19—C23 | −66.1 (5) |
| C19—C10—C11—C12 | −52.6 (7) | C9—C10—C19—C23 | 62.6 (5) |
| C20—C11—C12—C13 | −138.1 (6) | C11—C10—C19—C14 | 57.8 (4) |
| C10—C11—C12—C13 | 45.6 (9) | C9—C10—C19—C14 | −173.5 (3) |

*Hydrogen-bond geometry (Å, º)*

| *D*—H···*A* | *D*—H | H···*A* | *D*···*A* | *D*—H···*A* |
|---|---|---|---|---|
| O16—H16*O*···O1$^{i}$ | 0.91 (7) | 2.34 (7) | 3.194 (4) | 158 (6) |
| C6—H6···O2$^{ii}$ | 0.95 | 2.54 | 3.355 (6) | 144 |
| C8—H8···O16$^{iii}$ | 0.95 | 2.59 | 3.256 (5) | 127 |
| C16—H16···*Cg*2$^{iv}$ | 1.02 (5) | 2.69 (5) | 3.564 (5) | 146 (4) |
| C21—H21*A*···*Cg*1$^{v}$ | 0.98 | 2.96 | 3.923 (6) | 169 |

Symmetry codes: (i) *x*+1/2, −*y*+1/2, −*z*+1; (ii) −*x*+1, *y*+1/2, −*z*+3/2; (iii) *x*−1/2, −*y*+1/2, −*z*+1; (iv) −*x*−1, *y*+1/2, −*z*+3/2; (v) −*x*+1/2, −*y*+1, *z*−1/2.
